# Temperament and character traits in substance use disorder in Iran: a case control study

**DOI:** 10.1186/s40359-021-00647-x

**Published:** 2021-09-11

**Authors:** Najmeh Shahini, Ali Talaei, Zanireh Salimi, Moussalreza Adinepour Sarab, Shakiba Gholamzad, Ali Teimouri, Saeedeh Hajebi Khaniki, Mohammadzaman Kamkar

**Affiliations:** 1 Golestan Research Center of Psychiatry (GRCP), Golestan University of Medical Sciences, Gorgān, Iran; 2 Psychiatry and Behavioral Sciences Research Center, Mashhad University of Medical Sciences, Mashhad, Iran; 3 Educational Supervisor of Ibn Sina Hospital and Dr. Hejazi, Mashhad University of Medical Sciences, Mashhad, Iran; 4 Student Research Committee, Iran University of Medical Sciences, Tehran, Iran; 5 Student Research Committee, Mashhad University of Medical Sciences, Mashhad, Iran; 6 Student Research Committee, Department of Biostatistics, School of Health, Mashhad University of Medical Sciences, Mashhad, Iran

**Keywords:** Temperament, Character traits, Substance use disorder

## Abstract

**Background:**

Patients with Substance use disorder have distinct personality traits, they were high score in novelty seeking (NS) and sensation seeking and lower in Self-directedness and higher in Self-transcendence, so we aim to investigate the relationships of temperament and characteristics with related some variables such as substance of choice.

**Design and setting:**

A case–control study enrolling 70 Substance use disorder patients and 70 controls was conducted at Mashhad University of medical sciences.

**Methods:**

Using a case–control design, a group of 70 Substance use disorder patients and 70 controls was conducted at Mashhad university of medical sciences. All participation completed the 240 questions of Temperament and Character Inventory-Revised (TCI-R). Multivariate analysis of covariance (MANCOVA) was employed to compare the relationship between temperament and character traits and patterns of substance use.

**Results:**

The scores of reward dependence, persistence, self-directedness, cooperativeness, and self-transcendence were significantly lower in the case group compared to healthy individuals (*P* < 0.05). In contrast, the score of novel seeking was significantly higher in the case group (*P* < 0.05). On the other hand, harm avoidance was not significantly different between the two studied groups (*P* = 0.637).

**Conclusions:**

Higher NS in patients with substance use disorder is common and different traits, and temperaments would choose different substance combinations.

## Introduction

Substance use disorder is the third significant perplexity in the society of Iran after inflation and unemployment [1]. Substance use disorder is described by a cluster of cognitive, behavioral, and physiological symptoms caused by compulsive drug-seeking despite the significant substance-related problems based on the Diagnostic and Statistical Manual of Mental Disorders, Fifth Edition [2, 3].

Due to neurochemical stimulation of the continuous loops of drug-induced, addiction stimulates the brain's neuronal circuits that mediate reward, motivation to behavioral inflexibility, and disruption of self-control and compulsive drug intake [4]. In the US, estimated that 53.2 million people used substances in 2018. Based on the National Survey on Drug Use and Health, the abuser of Methamphetamine, Heroin, Methadone, and sedative was 1.1 million, 808,000, 256,000, and 751,000 [5]. According to Nikfarjam et al. study, the most common variety of substance use in Iran is opium, shire, crystal methamphetamine, cannabis, heroin/crack, stimulants (e.g., methamphetamine),and injecting substance with the prevalence of 1500, 660, 590, 470, 350, 300 and 280 per 100,000 individual respectively [6]. Despite many other countries, crack in Iran is a cocaine-free substance, and it's a combination of heroin, codeine, morphine, and others [7].

Temperament and characteristic traits are suggested by Cloninger. Temperament in childhood is consensually defined as early appearing and constitutionally based biological and self-regulation as exhibited in different contexts in response to stimulation. Besides child temperament is characterized by two core features: First, temperament is manifest in individual observable behaviors, and, second, temperament (contra, say, emotions) is hypothesized to be relatively stable [8]. Individual differences in temperament are measurable early in the development phase, and reflect individual differences in brain structures and overall function. They are less subject to change. The four temperament traits Are Harm avoidance (HA) is primarily an inhibitory inclination; individuals high in HA are pessimistic, fearful, shy away from novel stimuli, and are fatigable. Novelty seeking (NS) are individuals with a curious, impulsive, spendthrift, and. HA and NS correlate negatively but weakly, so that they are not opposite poles of a single temperament dimension, and are both high in many individuals with attention-deficit/hyperactivity disorder. Individuals high in reward dependence (RD) are sentimental, make close attachments, are highly sensitive to social cues, and are very dependent on social acceptance. Persistence temperament (P) are hardworking, will not easily be frustrated as they work toward a goal, and are perfectionistic.

There are three character traits. Self-directedness (SD) are responsible, goal-oriented, resourceful, self-accepting, and have good habits that help their chosen directions. cooperativeness (CO) are accepted others, are empathic and sympathetic, help others, and are guided by explicit pro-social values [9]. Individuals with self-transcendence (ST) have experience self-forgetfulness and flow, identify with groups or values beyond their existence, and are More spiritual and less materialistic [10].

The association of personality and temperament traits with substance use disorder has been documented [11, 12]. Comorbidity of personality disorders (PDs) and substance use disorders (SUDs) is common in clinical practice. Borderline PD and antisocial PD are particularly found to be associated with SUDs. The overall prevalence of PD ranges from 10 to 14.8% in the normal population and from 34.8 to 73.0% in patients treated for addictions. The prevalence of any PD is higher among patients with drug use disorder than alcohol use disorder. The co-morbidity with PD positively correlates with the severity of the SUD [11]. Certain temperamental traits played a significant role in the onset, formation, and continuation of drug dependency [13].

In summary, a high score in novelty seeking (NS) and sensation seeking (SS) is reported in many individuals with cocaine, alcohol, and heroin users [14–16]. Novelty seeking (NS) is a temperamental trait that is associated with high impulsivity exploratory behavior, extravagance and disorderliness. It is closely related to positive emotionality and sensation-seeking (SS). High NS (and related constructs) are associated with a compulsive drug-seeking behavior, SUDs and worse treatment outcomes in patients with an alcohol use disorder. It is hypothesized that alterations in central dopaminergic functioning explain the association between NS and SUDs [16]. So we are aware of a few study in to assess the relationship between personality features Influence one’s choice of drug [17, 18]. This study found several robust differences in temperament and character among substance use disorder. Our study is an attempt to replicate these findings using the new version of Character Inventory-Revised (TCI-R), which has 240 questions, in a different culture [7], and different socioeconomic setting in Iran.

## Material and methods

### Design

This was a case–control design in which data from Patients with Substance use disorder control subjects were collected in a convenience sample. All patients with substance use disorder were chosen randomly from patients with substance use disorder visiting Ibn-e-Sina hospital, Mashhad, Iran, in 2016–2018 and consulted by a psychiatrist. The control group was composed of age-unmatched and gender-matched subjects recruited from among individuals without substance use disorder.

Inclusion criteria were diagnosed with substance use disorder by DSM V criteria, and exclusion criteria were unwilling to the study and all subjects with comorbid Axis I disorders were also excluded in this study. Comorbid Axis I diagnosis was based on a clinical interview by a psychiatrist and DSM V criteria. The control group was composed of age-unmatched and gender-matched subjects recruited from among individuals without substance use disorder and all of them assessed by a psychiatrist.

### Subject

Patients aged between 18 and 55 years with substance use disorder were recruited.

In this study, we classified substance use disorder patients into five categories, including 1-Methadone, Sedative, Opium 2-Methadone, Amphetamine 3-Methadone, Sedative, Amphetamine 4-Methadone, Opium 5-Amphetamine, opium.

### Sample size

Acknowledging Moreira et al.’s study with a significance level of 0.05 and the test power of 80% to estimate the sample size, we considered 70 patients as a case and 70 individuals as healthy control [19].

### Assessments

At first, we asked the enrolled patients to fill an information form consist of demographic information, including age, sex, patterns of substance use, and educational degree levels.

Personality traits were assessed using the self-administrated Brazilian version of TCI-R consisting of 240 self-descriptive true/false items, assessing four temperament dimensions: NS (range 0–40; sign: positive; minimum significant score: N/A); HA (range 0–35; sign: positive; minimum significant score: N/A); RD (range 0–24; sign: positive; minimum significant score: N/A); P (range 0–8; sign: positive; minimum significant score: N/A); SD (range 0–44; sign: positive; minimum significant score: N/A); and three character dimensions: C (range 0–42; sign: positive; minimum significant score: N/A); and ST (range 0–33; sign: positive; minimum significant score: N/A) [20].

### Statistical analysis

We used SPSS software (version 20; SPSS Inc., Chicago, IL) for the statistical analysis. A *P* value of less than 0.05 was considered significant.

The description of continuous and categorical variables (nominal or ordinal) included mean, median, standard deviation, minimum, maximum frequency, and percent. The Homogeneity of groups in terms of demographic variables were assessed by chi-square or t-test. The Mann–Whitney test was used to check the differences in TCI-R and their domains between two groups or subgroups. Multivariate analysis of covariance (MANCOVA) was employed to assess the relationship between temperament and character traits and patterns of substance use.

## Results

In this study, 70 men with substance use disorder and 70 healthy individuals were examined. The mean age of the cased was 34.2 ± 7.8 years, with a range of 19 to 54 years, while the mean age of controls was 28.7 ± 9.1 years, which was significantly lower than cases (*P* < 0.0001). Moreover, the control group consisted of 11 females (15.7%) and 59 males (84.3%), whereas the studied cases were men (*P* = 0.001). The average age of onset of substance use was 19.37 ± 6.15 years. The majority of those surveyed cases were married (64.3%), unemployed (40.0%), and under-educated, which were significantly different from the control group (*P* < 0.05) (Table 1). Besides, 40% of individuals with substances use disorder have been taking substance daily for more than ten years. The most commonly used substances were opium (82.9%) and opium poppy (71.4%). Also, 80% of patients smoked, 64.3% consumed alcohol, and 60% used sedatives. In total, 64.3% of studied individuals were polysubstance dependent. Twenty-five percent of people had a history of psychological disorders, and 20% had a past medical history.

**Table 1:** Comparison of demographic variables between case and control groups

	Control (n = 70)	Case (n = 70)	*P* value*
Sex (male/female)	59/11	70/0	0.001
Age (years)	28.7 ± 9.1	34.2 ± 7.8	< 0.0001
Marital status			< 0.0001
Married	58 (82.9%)	16 (22.9%)	
Single	12 (17.1%)	45 (64.3%)	
Widow/divorced	0 (0%)	9 (12.9%)	
Education			< 0.0001
Secondary School	3 (4.3%)	30 (42.9%)	
Diploma	9 (12.9%)	28 (40.0%)	
Undergraduate	26 (37.1%)	12 (17.1%)	
Postgraduate	32 (45.7%)	0 (0%)	
Job			0.049
Full time	23 (32.9%)	15 (21.4%)	
Part time	32 (45.7%)	27 (38.6%)	
Unemployed	15 (21.4%)	28 (40.0%)	

Data were presented as Mean ± SD, frequency (%)

*Based on Chi-square test

### Comparison of case and control groups in terms of temperament and character traits

#### Univariate analysis

The scores of reward dependence, persistence, self-directedness, cooperativeness, and self-transcendence were significantly lower in the case group compared to healthy individuals (*P* < 0.05). In contrast, the score of novel seeking was significantly higher in the case group (*P* < 0.05). On the other hand, harm avoidance was not significantly different between the two studied groups (*P* = 0.637) (Table 2).

**Table 2:** Distribution of Temperament and character scores in case and control groups

	Control (n = 70)		Case (n = 70)		P-value*
	Mean ± SD	Median (min–max)	Mean ± SD	Median (min–max)	
HA	90.6 ± 9.3	88 (70–117)	90.9 ± 13.8	92 (59–129)	0.637
NS	96.4 ± 9.2	95.5 (84–122)	101.9 ± 11.7	103 (76–128)	0.002
RD	181.1 ± 8.2	180 (165–210)	99.8 ± 10.2	98.5 (73–127)	< 0.001
PS	108.8 ± 15.5	106.5 (78–152)	121.4 ± 12.0	122 (97–156)	< 0.001
SD	107.1 ± 9.6	106 (83–137)	124.1 ± 18.7	123 (80–164)	< 0.001
CO	101.6 ± 8.2	101 (84–119)	125.6 ± 11.9	124.5 (103–154)	< 0.001
ST	77.3 ± 10.9	77 (47–104)	90.5 ± 10.2	90 (72–111)	< 0.001
TCI-R	664.7 ± 58.2	662.5 (536–824)	754.3 ± 30.8	751 (696–840)	< 0.001

TCI-R, Temperament and Character Inventory-Revised; HA, Harm Avoidance; NS, Novel Seeking; RD, Reward dependence; PS, Persistence; SD, Self-directedness; CO, cooperativeness; ST, Self-transcendence

*Based on Mann–Whitney test

#### Multiple analysis

Since sex, age, marital status, and level of education were significantly different between groups, we run the multivariate analysis of covariance (MANCOVA) to adjust the effect of these variables considering the correlation between subscales of TCI-R. The score of RD (F (1,126) = 14.24, *P* < 0.001, η^2^ = 0.102), PS (F (1,126) = 6.91, *P* = 0. 01, η^2^ = 0.052), SD (F (1, 126) = 12.83, *P* < 0.001, η^2^ = 0.092), CO (F (1, 126) = 46.22, *P* < 0.001, η^2^ = 0.268) and ST (F (1, 126) = 11.99, *P* = 0.001, η^2^ = 0.087).

### Relationship of temperament and character traits with studied variables

#### Univariate analysis

**Fig. 1:**
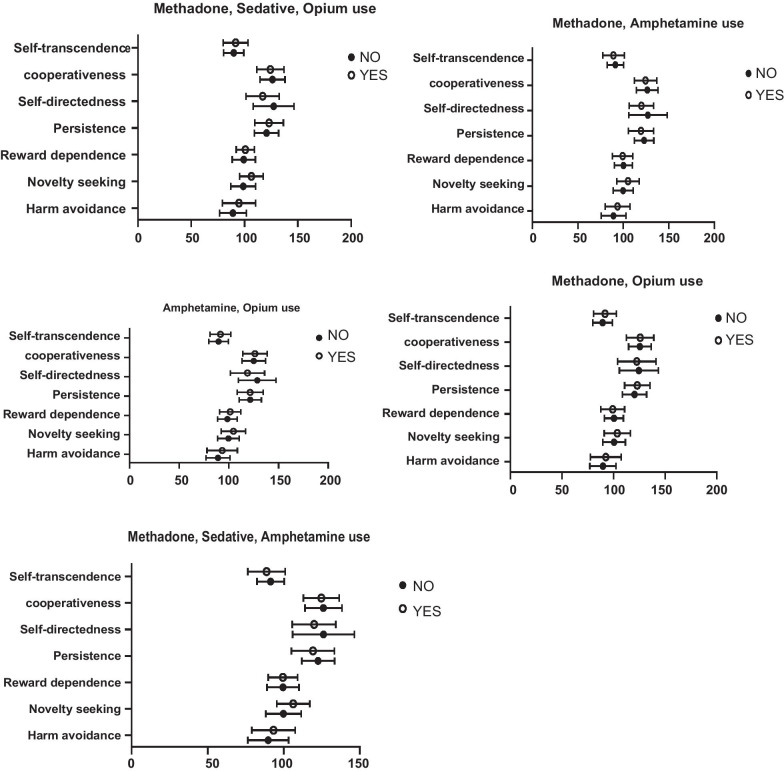
Temperament and character mean scores in relation to polysubstance dependence

*Methadone, Sedative, Opium:* 22 individuals (31.4%) were substance dependent in all three methadone, sedatives, and opium substances. Individuals with simultaneous use of methadone, sedative, or opium had an increase in NS scores as compared with those with no use of these three substances (F (1,68) = 5.17, *P* = 0.02, η^2^ = 0.071). SD scores were significantly much lower in individuals who had Methadone, Sedative, and Opium dependence (F (1,68) = 4.90, *P* = 0.03, η^2^ = 0.067). HA (F (1,68) = 2.64, *P* = 0.108, η^2^ = 0.037), RD (F (1,68) = 0.31, *P* = 0.58, η^2^ = 0.005), PS (F (1,68) = 0.56, *P* = 0.45, η^2^ = 0.008), CO (F (1,68) = 0.41, *P* = 0.53, η^2^ = 0.006), ST (F (1,68) = 0.48, *P* = 0.49, η^2^ = 0.007) were not significantly related to methadone, sedative or opium. Moreover, the total score of TCI-R was not significantly different between the mentioned two groups (*P* = 0.65) (Fig. 1).

*Methadone, Amphetamine: *Out of 70 studied individuals, 28(40%) had dependence in both methadone and amphetamine. None of TCI-R components, NS (F (1,68) = 3.83, *P* = 0.06, η^2^ = 0.053), HA (F (1,68) = 1.78, *P* = 0.18, η^2^ = 0.026), RD (F (1,68) = 0.13, *P* = 0.72, η^2^ = 0.002), PS (F (1,68) = 1.44, *P* = 0.23, η^2^ = 0.021), SD (F (1,68) = 2.5, *P* = 0.12, η^2^ = 0.036), CO (F (1,68) = 0.46, *P* = 0.49, η^2^ = 0.007), ST (F (1,68) = 0.71, *P* = 0.43, η^2^ = 0.010) were significantly associated with methadone and amphetamine dependence. Also, the total score of TCI-R was not significantly different between the mentioned two groups (*P* = 0.179) (Fig. 1).

*Methadone, Sedative, Amphetamine: *Overall, 23 individuals (32.9%) were dependent on all three substances of methadone, sedatives, and amphetamine. The NS score was significantly higher in those individuals who were dependent on methadone, sedatives, and amphetamine as opposed to others (F (1,68) = 4.84, *P* = 0.03, η^2^ = 0.066). However, HA (F (1,68) = 0.96, *P* = 0.31, η^2^ = 0.014), RD (F (1,68) = 0.03, *P* = 0.85, η^2^ = 0.000), PS (F (1,68) = 1.24, *P* = 0.27, η^2^ = 0.018), SD (F (1,68) = 1.70, *P* = 0.19, η^2^ = 0.024), CO (F (1,68) = 0.20, *P* = 0.65, η^2^ = 0.003), ST (F (1,68) = 1.04, *P* = 0.31, η^2^ = 0.015) were not significantly different in those who abuse methadone, sedative or amphetamine in comparison to others. To add more, the total score of TCI-R was not significantly different between the mentioned two groups (*P* = 0.649) (Fig. 1).

*Methadone, Opium: *Overall, 23 individuals (32.9%) were dependent on all three methadone, sedatives, and amphetamine substances. None of TCI-R components, NS (F (1,68) = 1.16, *P* = 0.28, η^2^ = 0.017), HA (F (1,68) = 0.71, *P* = 0.40, η^2^ = 0.01), RD (F (1,68) = 0.25, *P* = 0.62, η^2^ = 0.004), PS (F (1,68) = 0.86, *P* = 0.36, η^2^ = 0.012), SD (F (1,68) = 0.45, *P* = 0.51, η^2^ = 0.007), CO (F (1,68) = 0.009, *P* = 0.92, η^2^ = 0.000), ST (F (1,68) = 0.89, *P* = 0.35, η^2^ = 0.013) were significantly associated with methadone and opium dependence. Also, the total score of TCI-R was not significantly different between those dependent on methadone and opium compared to others (*P* = 0.777) (Fig. 1).

*Amphetamine, Opium: *In those 32 individuals who were amphetamine and opium dependent, sedatives and opium. Individuals with simultaneous use of opium and amphetamine had a decrease in SD scores compared to others (F (1,68) = 5.19, *P* = 0.03, η^2^ = 0.071) (Fig. 1).

*Age: *None of TCI-R components, except ST (F (1,68) = 5.74, *P* = 0.02, η^2^ = 0.078), were significantly associated with age. The older the individual, the higher the score of self-transcendence.

*Studies*: In the univariate analysis, there were no statistical differences between the individual with different levels of education and scores of TCI-R.

*Past Psychological history: *According to the result of MANCOVA, those patients with PPH had significantly higher scores of HA (F (1,68) = 4.0, *P* = 0.04, η^2^ = 0.056) and lower scores of ST (F (1,68) = 4.2, *P* = 0.03, η^2^ = 0.058).

*Alcohol dependence*: There was no statistical association between scores of TCI-R and alcohol dependence.

*Polysubstance dependence:* About 36% of the studied individual were polysubstance dependent in whom the scores of RD (F (1,68) = 8.9, *P* = 0.004, η^2^ = 0.116), PS (F (1,68) = 11.04, *P* = 0.001, η^2^ = 0.140) and ST (F (1,68) = 7.65, *P* = 0.007, η^2^ = 0.101) were significantly lower than others (Table 3).

**Table 3:** Distribution of Temperament and character scores in polysubstance dependents

	Polysubstance dependence				P-value
	Yes (n = 25)		No (n = 45)		
	Mean ± SD	Median (min–max)	Mean ± SD	Median (min–max)	
HA	94.5 ± 14.8	92 (69–129)	88.9 ± 12.8	93 (59–112)	0.477
NS	100.6 ± 10.7	101 (82–128)	102.7 ± 12.4	105 (76–125)	0.107
RD	95.2 ± 8.1	98 (73–108)	102.4 ± 10.4	101 (80–127)	0.004
PS	115.5 ± 9.7	118 (99–132)	124.8 ± 11.9	123 (97–156)	0.001
SD	123.7 ± 19.7	123 (91–164)	124.3 ± 18.3	123 (80–162)	0.900
CO	123.0 ± 12.3	122 (107–154)	127.1 ± 11.7	126 (103–152)	0.180
ST	86.2 ± 9.5	85 (72–111)	92.9 ± 9.8	92 (74–111)	0.007
TCI-R	738.6 ± 29.9	731 (696–840)	763.0 ± 27.9	763 (710–832)	-

*Based on MANCOVA

### Multiple analysis

To assess the effect of variables simultaneously, we performed multiple analysis of covariance considering age, studies, opium, sedative, amphetamine, methadone and alcohol dependence. The score of ST increased with age (F (1,61) = 7.25, *P* = 0.009, η^2^ = 0.106). Also, the NS scores increase significantly with a sedative (F (1,61) = 5.28, *P* = 0.02, η^2^ = 0.080) and amphetamine (F (1,61) = 7.59, *P* = 0.008, η^2^ = 0.111) dependence while SD score decreased in those individuals who were sedative dependent (F (1,61) = 5.67, *P* = 0.02, η^2^ = 0.085).

## Discussion

In this case–control study comparing 70 men with substance used disorder patients with 70 unmatched control, the combined scores for TCI were significantly different. Substance use disorder patients presented lower RD, PS, SD, CO, ST, and higher NS compare to the control group. On the other hand, the HA was not significantly different between the two studied groups. According to many studies, patients with substance use disorder presented higher HA and higher NS compared to healthy individuals [6, 13, 21–23]. However, some study assumed that NS was even lower in patients with substance use disorder than a control, for instance, Süleyman Can et al. study on substance abusers in the Turkish military population and concluded that NS and HA, and significantly lower scores for PE, SD, and CO were detected in substance abusers than in the controls [24].

Based on previous studies performed in Iran, including Abolghasemi et al. and Ketabi et al. study, both HA and NS were higher in substance disorder patients. In contrast, in the present study, despite the higher score of HA in the case group, the differences were statically meaningless [25, 26] but in clinical you found that high Harm Avoidance increases the risk of developing an addiction.

Following numerous studies, including Abolghasemi et al. and Ketabi et al. studies, in the present study, NS was significantly higher in the case compared to the healthy group [25, 26].

Patients with Amphetamine plus Methadone and Sedative use disorder showed higher NS than healthy control. In contrast, in patients with Amphetamine plus Methadone use disorder, TCI-R components were not significantly different from healthy control; this result maybe helps to aware the relationship between personality features that Influence one’s choice of drug.

Pournaghash et al. compared the TCI score of Amphetamine use disorder to opium used disorder and concluded that all TCI-R components were significantly higher in Amphetamine use disorder. In contrast, the present study showed that patients with Opium plus Methadone and Amphetamine plus Methadone use disorder were the same in all TCI scores. However, in Methadone plus Opium use disorder, SD was lower [23].

In our study, patients with a Polysubstance use disorder presented lower RD, PS, and ST, while in Koller et al., studies on NS and ST were higher in Polysubstance use disorder [27].

Controversy present in almost every research in TCI and addiction's field. These disagreements might exist because of diversity in population or even substantial differences worldwide.

In conclusion, higher novelty sicking in patients with substance use disorder is common and different traits, and temperaments would choose different substance combinations.

## Limitation

Our study is retrospective, that is, personality factors are studied in individuals who are already addicted, so that the causal direction of the findings is uncertain. Optimally the causal hypotheses of the kind made are more reliable if found in prospective follow-up studies. other limitations of the study is the small sample size, In future studies, we strongly suggested that future studies design to investigate the effect of this method with higher sample size and prospective follow-up studies.

## Data Availability

The data for the current study will not be shared publicly as participants were informed at the time of providing consent that only researchers involved in the project would have access to the information they provided.
